# Supplementary material for: Predation and fragmentation portrayed in the statistical structure of prey time series
Source: BMC Ecol. 2009 May 6;9:10. doi: 10.1186/1472-6785-9-10 (PMC2689204; doi:10.1186/1472-6785-9-10)
Supplement: Additional file 2 — Voles and related classes ODDox Documentation. ODDox documentation of the agent-based model (ALMaSS) applied by Hendrichsen et al. The documentation is started by activating main.html. [file 1472-6785-9-10-S2.zip › Vole_ODDox/_genetic_material_8_h.html]

ALMaSS ODDox: GeneticMaterial.H File Reference

- Main Page
- Related Pages
- Classes
- Files

# GeneticMaterial.H File Reference

---

## Detailed Description

**GeneticMaterial.h This file contains the headers for the genetic material classes**   

by Chris J. Topping   
Version of 1st Nov 2000   
All rights reserved.   
  
With additions as noted in:   
Doxygen formatted comments in May 2008   

|  |  |
| --- | --- |
|  |  |
| Classes | |
| class | AlleleFreq |
|  | Class to handle statistics and constructs based on allele frequencies. More... |
| class | GeneticMaterial |
|  | Class for the genetic material optionally carried by animals in ALMaSS. More... |
| class | GeneticMaterial1616 |
| Typedefs | |
| typedef unsigned int | uint32 |

---

## Typedef Documentation

typedef unsigned int uint32

---

Generated on Thu Jan 22 14:13:45 2009 for ALMaSS ODDox by 
 1.5.6
